# Gender differences in quality of life among community-dwelling older adults in low- and middle-income countries: results from the Study on global AGEing and adult health (SAGE)

**DOI:** 10.1186/s12889-020-8212-0

**Published:** 2020-01-28

**Authors:** Kyung Hee Lee, Hanzhang Xu, Bei Wu

**Affiliations:** 1 Yonsei University College of Nursing, 50-1 Yonsei-Ro, Seodaemun-Gu, Seoul, 03722 South Korea; 2 Mo-Im Kim Nursing Research Institute, Seoul, South Korea; 3 Duke Department of Family Medicine and Community Health and Duke University School of Nursing, Durham, NC USA; 4 New York University Rory Meyers College of Nursing, New York, NY USA

**Keywords:** Quality of life, Gender inequality, Older adults, Low- and middle-income country

## Abstract

**Background:**

Quality of life (QoL) is an important component of individuals’ general well-being, particularly in older adults. However, factors influencing QoL among older adults in low- and middle-income countries (LMICs) have not been fully examined. Furthermore, the role of gender differences in relation to QoL in multiple LMICs has also not been examined in detail.

**Methods:**

This study used data from the World Health Organization’s Study on global AGEing and adult health (SAGE), Wave-1. Based on a literature review of existing works, a set of variables—an independent variable and covariates—were selected. The study sample consisted of 33,019 participants aged 50 years and above from China, Ghana, India, Russia, and South Africa. Multivariate linear regression models were estimated with the World Health Organization QoL scores as the dependent variable. To preserve the analytical sample size, multiple imputation was used to account for missing data.

**Results:**

The results showed that generally, male older adults reported a better QoL than female older adults across all of the countries. The associations between QoL and sociodemographic factors, health-related factors, and social support factors among older adults differed according to country.

**Conclusions:**

This study provides a better understanding of QoL among older adults in LMICs, which can help prepare LMICs to better address the QoL of older adults. The results of this study can be used to develop programs to promote better living standards and services to reduce gender disparities and ultimately, to improve the QoL among older adults in LMICs.

## Background

In the period from 2010 to 2015, the United Nations Population Division confirmed that the global life expectancy at birth was 71 years and projected this life expectancy to increase continuously around the world, particularly in low- and middle-income countries (LMICs) [1]. According to the World Health Organization (WHO), by 2050, 80% of all older adults will live in LMICs [2]. Population aging poses great challenges to society concerning—for example—health care, caregiving, and a suitable pension system. This particularly applies to developing and underdeveloped countries that often have limited resources. As the global life expectancy increases and people live longer, quality of life (QoL) is one of the most important indicators for modern society.

The World Health Organization’s Quality of Life assessment (WHOQoL) Group defined QoL as “an individuals’ perception of their position in life in the context of culture and value systems in which they live and in relation to their goals, expectations, standard, and concerns” (p.1570) [3]. The multidimensional concept of QoL is a valuable measure for understanding overall subjective well-being, which also strongly affects older adults’ clinical decisions. Moreover, QoL measures can be utilized to monitor disease progress, evaluate treatment, and prioritize problems in clinical practice [4]. It is therefore important to determine overall QoL and its related factors among older adults.

Most existing studies concerning QoL and its influencing factors in older adults were conducted in developed countries. Therefore, the findings of these studies are likely to differ from the findings of studies conducted in LMICs, largely due to different levels of social and economic development, health care systems, and national life expectancy [5]. There are very few studies that have examined the influencing factors of QoL among community-dwelling older adults in LMICs and most of these studies only examined the association between a small number of independent variables and QoL in one specific country [6, 7]. Furthermore, there are previous studies that have focused not on overall QoL, but on specific domains, for example oral health-related QoL [8], work-related QoL [9], or vision-related QoL [10]. Certain studies have also focused on specific populations, for example stroke survivors [11], patients with heart failure [12], or migrant workers [13].

Gender difference is another important factor of QoL in LMICs, as gender plays an essential role in decision-making as well as the perception of health across countries and cultures. Although there have been reports of worse health-related QoL among women in developed countries [14], it is still not fully understood what role gender plays in overall QoL in multiple LMICs.

Examining the similarities and differences in QoL across LMICs is therefore essential, which is why the current study included five LMICs (i.e., China, India, Russia, South Africa, and Ghana) that represents approximately half of the world’s older adult population. Since these five countries all have different socioeconomic statuses, represent a wide range of racial and ethnic groups, and are geographically situated in different regions, they offer a wide representation of LMICs. Examining the overall QoL among older adults from nationally representative samples in LMICs is necessary to identify both universal and country-specific factors associated with QoL in LMICs, to prepare LMICs to better address the QoL of older adults, and to understand the relationship between aging and well-being. Based on the above, the purpose of this study is to identify the influencing factors on QoL in community-dwelling older adults in LMICs and to define the role of gender in relation to QoL. This study aims to provide empirical evidence that could assist in developing intervention programs for improving the QoL of older adults, consequently providing global public benefit to the aging population in LMICs.

## Methods

### Study design and data sample

This study used data from the WHO’s Study on global AGEing and adult health (SAGE), Wave-1 (2007–2010). The data were originally collected in six LMICs—China, Ghana, India, Mexico, the Russian Federation, and South Africa—to better understand the health and well-being of older adults through nationally representative samples. SAGE is designed as a multi-wave panel study. Multistage cluster sampling methods were used; the original sample consists of 35,334 people aged 50 years or older who participated in the SAGE Wave-1 initiative. Face-to-face interviews were conducted using a standardized survey instruments, set of methods, interviewer training and translation protocols in all countries. A more detailed description of the SAGE Wave-1 data has previously been published [15]. The final sample for this study comprised 33,019 people aged 50 or older in five countries, after we excluded the data from Mexico due to substantial missing values (49.7% of data).

### Outcomes of interest

The main outcome variable for this study is QoL. QoL was assessed using the 8-item WHOQoL instrument [16]. The 8-item WHOQoL—a shortened version of the WHOQoL-BREF—comprised two items from each domain of the WHOQoL-BREF (i.e., physical, psychological, environmental, and social). Participants answered each question rated on a five-point Likert scale from 1 (not at all) to 5 (completely). The overall QoL score was determined by a simple summation of the scores of the eight items and then rescaling the score from 0 to 100, where a higher score indicated a higher QoL. Good internal consistencies (0.72–0.85) [16, 17] and acceptable convergent validity with WHOQoL-BREF (0.61–0.77) [17] were reported across the five countries.

### Independent variable

Gender was assessed as the independent variable by recording the gender of the participant (male = 0, female = 1).

### Covariates

The covariates consisted of demographic variables (i.e., age, education, health insurance, income, and living environment), health-related variables (i.e., cognitive function, physical function, presence of comorbidities) and social support variables (i.e., marital status, family support, community support, social cohesion index, and living arrangements).

Sociodemographic variables included age (continuous variable), education (0 = less than primary, 1 = primary only, 2 = secondary only, 3 = high school only, 4 = college and above), and health insurance (no = 0, yes = 1). Furthermore, standardized income (continuous, provided by SAGE, with a higher score of standardized income indicating a higher income status) and living environment was assessed by a summary scale based on three dichotomized indicators related to an individual’s living environment (i.e. hard floor, piped drinking water, and durable walls). The total score ranged from 0 to 3, with higher scores indicating a better living environment.

Cognitive function was measured by five tests: forward and backward digital span tests, verbal fluency, immediate recall, and delayed recall. This set of cognition tests captured several aspects of cognitive function, including working memory. First, a z-score was generated from each test before a global cognition score was calculated by averaging the z-scores. Higher z-scores indicated better cognitive function. Physical function was assessed by using the 12-item version of the World Health Organization Disability Assessment Schedule (WHODAS) 2.0 [18]. This test is a brief assessment tool to measure physical functional limitations cross-culturally. Research examining the psychometric properties of the test supported the construct validity of the one-factor solution with various samples [19–21] and a strong internal consistency [21]. A higher WHODAS 2.0 score indicates poorer physical function. Comorbidity was defined according to the presence of arthritis (no = 0, yes = 1), hypertension (no = 0, yes = 1), and diabetes (no = 0, yes = 1).

Marital status (not married = 0, married = 1) was included as a social support variable. Received social support was defined as family support and community support. The SAGE Household survey was conducted to determine whether the participants received any financial or in-kind support from 1) family members or 2) the community. Two dummy variables were created if the respondents received any family or community support. The social cohesion index consisted of 9 questions related to the frequency of taking part in various social activities, for example attending religious services or having friends over [22]. The total social cohesion index score ranged from 9 to 45, with higher scores indicating better social cohesion. The living arrangements variable was created as a dichotomized variable if participants reported a household size of one. Lastly, a country variable was included (101 = China, 106 = India, 102 = Russia, 103 = South Africa, 104 = Ghana).

### Statistical analysis

The sample characteristics of the study participants were determined, and comparisons according to country were calculated with chi-square and ANOVA tests. Moreover, a post hoc analysis for group differences was performed using the Bonferroni correction. The *p*-values were based on 2-tailed tests and can be considered statistically significant at *p* < .05. Overall 8.4% of the study participants had missing data in their QoL measure. The percentage of missing values across all covariates ranged from 1% (self-reported arthritis) to 6% (physical function). To preserve the analytical sample size, multiple imputation (mi impute mvn command in STATA) was used to account for missing data (5 imputations). A preliminary analyses produced results similar to that of the multiple imputation when using listwise deletion to address missing data.

Next, multivariate linear regression models were estimated to examine the factors influencing QoL. The first set of analyses estimated differences in QoL while adjusting for different countries. The second set of analyses estimated differences in QoL while adjusting for sociodemographic variables. The third set of analyses was based on the second set of analyses while adding health-related variables into the model. Next, the fourth set of analyses included all of the factors mentioned above as well as social support factors. Because we observed significant differences in QoL as well as other sample characteristics across the five countries, we then stratified the analyses according to country (Table 3). Next, we performed further analyses to examine the influencing factors on QoL according to gender (Tables 4 and 5). Standardized coefficient estimates were presented to assist in identifying the most influential factors. Survey weights were used in the descriptive analyses to adjust for the sampling design. For the multivariate analyses, results from the unweighted models were presented, as all multivariate analyses included variables used in the sampling weights (e.g., age and gender). This was done because including survey weights may produce biased estimates and inflated standard errors [23]. The analyses in this study were conducted using Stata version 14.2.

## Results

**Fig. 1:**
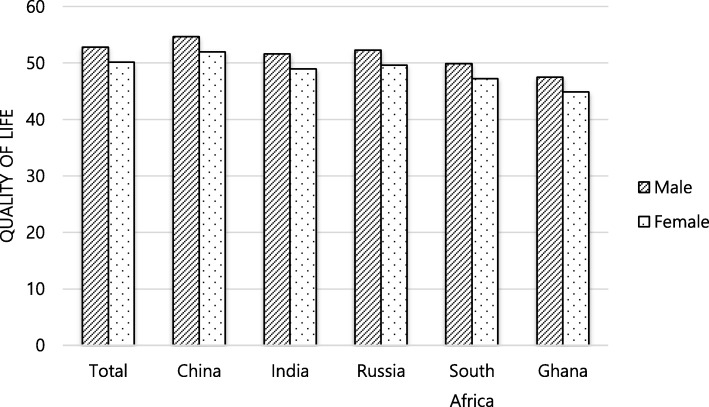
Quality of Life According to Country and Gender

This study used data from 33,019 participants aged 50 and older from five LMICs (Table 1). The univariate analyses showed that the QoL, sociodemographic factors, comorbidities, and social support differed significantly among the five sample countries. Furthermore, male participants consistently reported higher QoL scores than female participants across all five countries and the QoL scores of both male and female participants from China were the highest (Fig. 1).

**Table 1:** Sample Characteristics

Variables	Total *N* = 30,825	China *N* = 12,718	India *N* = 6534	Russia *N* = 3795	South Africa *N* = 3538	Ghana *N* = 4240	*p*
Age							<.001
50–59	46.6	45.2	48.6	45.5	49.4	40.0	
60–69	30.0	31.9	30.9	24.5	31.1	27.3	
70–79	18.1	18.6	16.0	21.5	14.0	23.1	
80 and above	5.3	4.3	4.5	8.5	5.5	9.6	
Female	52.0	50.2	49.0	60.9	56.1	47.7	<.001
Education							<.001
Less than primary	41.8	41.5	61.2	2.0	48.8	64.2	
Primary only	15.7	21.3	14.8	5.4	22.6	11.0	
Secondary only	16.0	19.9	10.2	20.4	14.2	4.1	
High school only	19.1	12.7	8.6	53.9	8.5	17.1	
College and above	7.4	4.5	5.1	18.4	5.9	3.6	
Married	75.7	85.4	76.9	58.6	55.6	59.2	<.001
Income	0.3 (0.6)	0.0 (0.5) ^abc^	0.7 (0·4)^adef^	0.0 (0.3)^dgh^	0.5 (1.0)^begi^	0.3 (1.4)^cfhi^	<.001
Living environment	2.2 (1.1)	2.6 (0.7) ^abcd^	1.4 (0·8) ^aef^	2.8 (0.3)^begh^	2.4 (1.8)^cfgi^	1.5 (2.8)^dhi^	<.001
Living arrangements	10.0	9.8	1.8	25.1	15.8	9.3	<.001
Medical insurance	56.6	89.8	3.9	99.6	20.3	38.1	<.001
Arthritis	22.1	22.0	18.2	30.1	24.8	14.0	<.001
Hypertension	47.0	56.4	27.9	60.2	71.5	56.0	<.001
Diabetes	6.8	6.6	6.9	7.0	9.0	3.8	<.001
Family support	27.2	35.4	26.3	14.1	9.7	43.3	<.001
Community support	3.9	2.4	6.7	1.9	1.9	3.3	<.001
Cognitive function	− 0.0 (1.0)	0.3 (1.0) ^abc^	−0.5 (0.6) ^adef^	0.5 (0.9) ^dgh^	−0.0 (1.7) ^begi^	− 0.2 (2.6) ^cfhi^	<.001
WHODAS	14.4 (16.2)	6.6 (10.4) ^abcd^	22.5 (13.3) ^aefg^	14.1 (11.7)^behi^	17.3 (33.5)^cfh^	18.5 (53.7)^dgi^	<.001
QoL	51.4 (12.3)	53.3 (12.1) ^abcd^	50.3 (9.5) ^aef^	50.7 (9.5) ^bg^	48.4 (23.1) ^ceh^	46.2 (40.8)^dfgh^	<.001

Data are % or mean (SD). ^a-i^ Groups with same letter are significantly different according to Bonferroni post hoc test

### Influencing factors on QoL among older adults in all five LMICs

The multivariate regression analyses (Table 2) showed that QoL differed significantly from country to country (Model 1). The full model (Model 4) showed that four sociodemographic factors (age, marital status, insurance, and income), comorbidity (cognitive function, physical function, arthritis, and diabetes), and social support (family and community support) were significantly related to QoL in all five countries. A comparison based on the magnitude of the effect across all factors revealed that income (β = 5.06), arthritis (β = − 2.39), and diabetes (β = − 2.31) were the top three influencing factors on QoL when considering all five countries.

**Table 2:** QoL influencing factors among older adults in five LMICs

	Model 1	Model 2	Model 3	Model 4
	*β* (SE)	*β* (SE)	*β* (SE)	*β* (SE)
Country (Ref: China)				
India	−3.42 (0.18)^†^	−6.69 (0.33)^†^	0.62 (0.31)^*^	−0.03 (0.31)
Russia	−3.36 (0.22)^†^	−5.34 (0.24)^†^	− 0.97 (0.22)^†^	−1.15 (0.22)^†^
South Africa	−3.71 (0.23)^†^	−6.63 (0.29)^†^	−2.55 (0.26)^†^	−3.70 (0.28)^†^
Ghana	−6.66 (0.21)^†^	−7.64 (0.27)^†^	−3.44 (0.25)^†^	−5.40 (0.28)^†^
Age group (Ref: 50–59)				
60–69	−1.93 (0.16)^†^	− 1.32 (0.16)^†^	0.42 (0.14)^†^	0.55 (0.14)^†^
70–79	−4.06 (0.18)^†^	−3.02 (0.18)^†^	1.00 (0.16)^†^	1.28 (0.17)^†^
80 and above	−7.49 (0.29)^†^	−5.96 (0.28)^†^	1.56 (0.26)^†^	2.14 (0.27)^†^
Female	−2.34 (0.14)^†^	−1.80 (0.13)^†^	−0.44 (0.12)^†^	− 0.03 (0.13)
Education (Ref: Less than primary)				
Primary only		0.43 (0.20)^*^	−0.21 (0.18)	−0.21 (0.17)
Secondary only		1.08 (0.21)^†^	−0.09 (0.19)	− 0.07 (0.19)
High school only		2.27 (0.23)^†^	−0.05 (0.21)	−0.08 (0.21)
College and above		4.22 (0.31)^†^	0.87 (0.28)^†^	0.68 (0.28)^*^
Health insurance		0.75 (0.21)^†^	0.57 (0.19)^†^	0.47 (0.19)^*^
Income		6.41 (0.18)^†^	5.16 (0.16)^†^	5.06 (0.16)^†^
Living environment		0.57 (0.09)^†^	0.56 (0.08)^†^	0.64 (0.08)^†^
Cognitive function			1.02 (0.08)^†^	0.89 (0.08)^†^
Physical function			−0.36 (0.00)^†^	−0.35 (0.00)^†^
Arthritis			−2.32 (0.15)^†^	− 2.39 (0.14)^†^
Hypertension			0.02 (0.12)	−0.01 (0.12)
Diabetes			−2.35 (0.23)^†^	−2.31 (0.23)^†^
Married				1.05 (0.16)^†^
Family support				0.09 (0.13)
Community support				−0.52 (0.31)
Social cohesion index				0.23 (0.01)^†^
Living arrangements				1.23 (0.21)^†^

Note. ^*^ < 0.05; ^†^ < 0.01

### Influencing factors on QoL among older adults according to country

When considering the five countries separately, the multivariate regression analyses showed associations between QoL and sociodemographic factors, health-related factors, and social support factors (Table 3). QoL was significantly associated with income, cognitive function, physical function, and the presence of arthritis and diabetes across the countries. Living environment and community support had a positive influence on QoL across all countries except Ghana. Family support was positively associated with QoL only in South Africa, while education did not have a significant influence on QoL across all five countries.

**Table 3:** QoL influencing factors among older adults according to country

	China	India	Russia	South Africa	Ghana
	*β* (SE)	*β* (SE)	*β* (SE)	*β* (SE)	*β* (SE)
Age group (Ref: 50–59)					
60–69	0.83 (0.22)^†^	−0.36 (0.28)	1.21 (0.43)^†^	1.58 (0.38)^†^	−0.24 (0.39)
70–79	2.25 (0.27)^†^	−0.54 (0.37)	2.41 (0.51)^†^	2.62 (0.48)^†^	−0.42 (0.46)
80 and above	4.05 (0.45)^†^	−0.27 (0.62)	4.93 (0.77)^†^	3.92 (0.71)^†^	−1.33 (0.63)^*^
Female	−0.42 (0.19)^*^	0.73 (0.29)^*^	−0.30 (0.37)	0.71 (0.37)	−0.14 (0.40)
Education (Ref: Less than primary)					
Primary only	0.14 (0.25)	−0.70 (0.38)	1.16 (1.15)	− 0.72 (0.43)	− 0.04 (0.53)
Secondary only	−0.16 (0.27)	−0.71 (0.46)	1.86 (1.07)	0.88 (0.54)	1.22 (0.82)
High school only	−0.10 (0.32)	0.06 (0.51)	1.58 (1.05)	1.01 (0.80)	−0.71 (0.48)
College and above	0.69 (0.47)	1.15 (0.65)	1.79 (1.11)	1.62 (0.92)	0.14 (0.90)
Health insurance	0.29 (0.29)	−1.20 (0.59)^*^	1.92 (2.60)	0.51 (0.44)	1.29 (0.33)^†^
Income	4.98 (0.25)^†^	4.26 (0.35)^†^	3.78 (0.50)^†^	5.66 (0.47)^†^	6.12 (0.47)^†^
Living environment	0.96 (0.15)^†^	0.58 (0.14)^†^	1.03 (0.38)^†^	1.29 (0.22)^†^	−0.44 (0.23)
Cognitive function	0.63 (0.11)^†^	0.93 (0.21)^†^	0.92 (0.21)^†^	1.09 (0.19)^†^	1.26 (0.22)^†^
Physical function	−0.40 (0.01)^†^	−0.31 (0.01)^†^	− 0.37 (0.02)^†^	−0.31 (0.01)^†^	− 0.36 (0.01)^†^
Arthritis	−2.78 (0.22)^†^	−2.31 (0.33)^†^	−2.98 (0.37)^†^	−1.60 (0.40)^†^	−1.05 (0.46)^*^
Hypertension	0.37 (0.18)^*^	0.13 (0.26)	−1.55 (0.34)^†^	0.47 (0.36)	−0.19 (0.31)
Diabetes	−3.24 (0.37)^†^	−1.58 (0.48)^†^	−2.01 (0.59)^†^	−1.67 (0.55)^†^	− 1.73 (0.80)^*^
Married	1.61 (0.30)^†^	0.63 (0.31)^*^	1.69 (0.47)^†^	1.05 (0.38)^†^	0.50 (0.40)
Family support	0.31 (0.21)	0.02 (0.27)	−0.71 (0.42)	1.27 (0.49)^†^	−0.08 (0.34)
Community support	−2.32 (0.67)^†^	−1.04 (0.44)^*^	2.93 (1.08)^†^	3.26 (1.30)^*^	−0.12 (0.87)
Social cohesion index	0.28 (0.03)^†^	0.31 (0.03)^†^	0.38 (0.04)^†^	0.05 (0.03)	0.20 (0.02)†
Living arrangements	1.65 (0.35)^†^	−1.59 (0.96)	1.67 (0.52)^†^	0.95 (0.52)	1.96 (0.54)†

Note. ^*^ < 0.05; ^†^ < 0.01

### Influencing factors on QoL among older adults according to gender

Tables 4 and 5 show the QoL influencing factors according to gender. Among the male participants, income, cognitive and physical function, and living environment were significantly associated with QoL among older adults in all five countries. Among the female participants, income and cognitive and physical function were significantly related to QoL in all five countries.

**Table 4:** QoL influencing factors among male older adults

	Total	China	India	Russia	South Africa	Ghana
	*β* (SE)	*β* (SE)	*β* (SE)	*β* (SE)	*β* (SE)	*β* (SE)
Country (Ref: China)						
India	−0.44 (0.45)					
Russia	−0.93 (0.33)^†^					
South Africa	−4.46 (0.41)^†^					
Ghana	−5.25 (0.40)^†^					
Age group (Ref: 50–59)						
60–69	0.26 (0.20)	0.57 (0.32)	−0.87 (0.39)^*^	1.79 (0.70)^*^	1.07 (0.58)	−0.19 (0.51)
70–79	1.08 (0.24)^†^	1.86 (0.38)^†^	−1.11 (0.50)^*^	3.53 (0.87)^†^	3.34 (0.76)†	−0.26 (0.62)
80 and above	2.33 (0.40)^†^	4.36 (0.66)^†^	0.05 (0.86)	8.66 (1.41)^†^	2.06 (1.13)	−1.00 (0.84)
Education (Ref: Less than primary)						
Primary only	−0.19 (0.25)	0.04 (0.36)	−0.61 (0.48)	1.52 (2.82)	−0.96 (0.79)	−0.08 (0.68)
Secondary only	−0.06 (0.27)	−0.03 (0.39)	− 0.95 (0.53)	1.50 (2.72)	1.00 (0.93)	1.58 (0.94)
High school only	−0.14 (0.28)	−0.40 (0.46)	− 0.04 (0.59)	2.72 (2.69)	0.96 (1.18)	−1.01 (0.58)
College and above	0.51 (0.37)	0.27 (0.61)	0.97 (0.75)	2.03 (2.75)	1.57 (1.23)	0.62 (1.05)
Health insurance	0.28 (0.26)	0.17 (0.44)	−0.95 (0.73)	0.97 (3.95)	0.38 (0.66)	0.72 (0.45)
Income	4.88 (0.23)^†^	4.74 (0.36)^†^	3.76 (0.49)^†^	3.19 (0.81)^†^	5.82 (0.72)^†^	6.35 (0.64)^†^
Living environment	0.73 (0.12)^†^	1.10 (0.21)^†^	0.81 (0.20)^†^	1.51 (0.63)^*^	1.43 (0.36)^†^	−0.62 (0.30)^*^
Cognitive function	0.97 (0.11)^†^	0.78 (0.16)^†^	1.18 (0.27)^†^	0.82 (0.34)^*^	1.07 (0.31)^†^	1.27 (0.29)^†^
Physical function	−0.36 (0.01)^†^	− 0.41 (0.02)^†^	− 0.32 (0.01)^†^	−0.42 (0.03)^†^	− 0.33 (0.02)^†^	−0.37 (0.02)^†^
Arthritis	−2.46 (0.22)^†^	−3.41 (0.34)^†^	−1.84 (0.47)^†^	−2.23 (0.68)^†^	− 1.09 (0.69)	− 1.92 (0.69)^†^
Hypertension	0.02 (0.17)	0.09 (0.27)	0.30 (0.37)	−1.68 (0.57)^†^	0.45 (0.54)	0.42 (0.42)
Diabetes	−2.08 (0.35)^†^	−2.91 (0.56)^†^	−1.19 (0.64)	− 2.66 (1.23)^*^	− 1.45 (0.92)	−2.42 (1.16)^*^
Married	0.46 (0.27)	1.19 (0.52)^*^	−1.14 (0.53)^*^	0.40 (1.12)	0.53 (0.63)	1.16 (0.62)
Family support	−0.02 (0.20)	0.28 (0.30)	−0.24 (0.39)	−1.33 (0.78)	1.07 (0.79)	0.05 (0.47)
Community support	−0.76 (0.46)	−2.47 (1.01)^*^	−1.03 (0.60)	3.21 (2.22)	3.87 (2.10)	−0.54 (1.32)
Social cohesion index	0.22 (0.02)†	0.28 (0.04)^†^	0.32 (0.04)†	0.37 (0.08)†	0.04 (0.05)	0.15 (0.03)^†^
Living arrangements	0.61 (0.35)	1.27 (0.58)^*^	−2.55 (2.09)	0.21 (1.25)	1.21 (0.78)	1.95 (0.75)^†^

Note. ^*^ < 0.05; ^†^ < 0.01

**Table 5:** QoL influencing factors among female older adults

	Total	China	India	Russia	South Africa	Ghana
	*β* (SE)	*β* (SE)	*β* (SE)	*β* (SE)	*β* (SE)	*β* (SE)
Country (Ref: China)						
India	0.56 (0.44)					
Russia	−1.24 (0.31)^†^					
South Africa	− 2.95 (0.38)^†^					
Ghana	−5.44 (0.39)^†^					
Age group (Ref: 50–59)						
60–69	0.88 (0.19)^†^	1.09 (0.30)^†^	0.30 (0.41)	0.83 (0.56)	2.05 (0.51)†	−0.22 (0.62)
70–79	1.60 (0.23)^†^	2.67 (0.37)^†^	0.28 (0.57)	1.75 (0.63)†	2.32 (0.62)†	−0.52 (0.68)
80 and above	2.22 (0.37)^†^	3.88 (0.64)^†^	−0.36 (0.94)	3.33 (0.90)†	5.29 (0.94)†	−1.60 (0.95)
Education (Ref: Less than primary)						
Primary only	−0.19 (0.26)	0.29 (0.36)	−0.80 (0.60)	0.89 (1.27)	−0.46 (0.63)	0.10 (0.83)
Secondary only	−0.03 (0.28)	−0.30 (0.38)	− 0.21 (0.87)	2.08 (1.16)	0.85 (0.71)	0.50 (1.69)
High school only	0.07 (0.32)	0.21 (0.45)	−0.04 (1.01)	1.04 (1.16)	1.05 (1.08)	0.11 (0.84)
College and above	0.98 (0.44)^*^	1.33 (0.78)	0.99 (1.38)	1.77 (1.25)	1.46 (1.31)	−0.46 (1.76)
Health insurance	0.65 (0.25)^*^	0.40 (0.37)	−1.73 (0.97)	2.32 (3.30)	0.53 (0.62)	1.80 (0.50)^†^
Income	5.17 (0.22)^†^	5.21 (0.35)^†^	4.68 (0.50)^†^	4.13 (0.64)^†^	5.44 (0.60)^†^	5.96 (0.70)^†^
Living environment	0.58 (0.11)^†^	0.84 (0.21)^†^	0.41 (0.21)^*^	0.76 (0.47)	1.22 (0.29)^†^	−0.28 (0.34)
Cognitive function	0.83 (0.11)^†^	0.51 (0.15)^†^	0.73 (0.28)^*^	0.97 (0.27)^†^	1.15 (0.25)^†^	1.23 (0.34)^†^
Physical function	−0.34 (0.01)^†^	−0.40 (0.01)^†^	− 0.31 (0.01)^†^	−0.35 (0.02)^†^	− 0.30 (0.01)^†^	−0.36 (0.02)^†^
Arthritis	−2.34 (0.19)^†^	−2.34 (0.28)^†^	−2.56 (0.46)^†^	−3.38 (0.43)^†^	−1.79 (0.50)^†^	−0.42 (0.63)
Hypertension	−0.04 (0.17)	0.62 (0.25)^*^	−0.07 (0.38)	−1.43 (0.43)^†^	0.44 (0.48)	−0.83 (0.47)
Diabetes	−2.46 (0.31)^†^	−3.57 (0.49)^†^	−1.95 (0.72)^†^	−1.84 (0.68)^†^	− 1.69 (0.70)^*^	−1.03 (1.13)
Married	1.50 (0.20)^†^	1.92 (0.38)^†^	1.62 (0.40)^†^	1.89 (0.53)^†^	1.44 (0.48)^†^	0.02 (0.54)
Family support	0.20 (0.18)	0.31 (0.28)	0.26 (0.39)	−0.53 (0.51)	1.45 (0.63)^*^	−0.22 (0.48)
Community support	−0.32 (0.42)	−2.17 (0.89)^*^	−1.01 (0.63)	2.72 (1.25)^*^	3.17 (1.66)	0.09 (1.19)
Social cohesion index	0.25 (0.02)^†^	0.28 (0.04)^†^	0.30 (0.04)^†^	0.38 (0.06)^†^	0.07 (0.04)	0.26 (0.04)^†^
Living arrangements	1.66 (0.27)^†^	1.90 (0.45)^†^	−1.10 (1.10)	2.04 (0.57)^†^	0.72 (0.72)	2.18 (0.79)^†^

Note. ^*^ < 0.05; ^†^ < 0.01

Concerning marital status, being married was significantly and positively associated with QoL among female participants from all countries, except for Ghana. However, this association was only significant for male participants from China and India. Comorbidities—specifically diabetes or arthritis—were negatively associated with QoL among female participants in all countries, except for Ghana. A similar association was found in male participants from China, Russia, and Ghana. Male participants who lived alone reported a higher QoL than those who lived with other family members in China and Ghana, whereas this association was significant among female participants in China, Russia, and Ghana. Family support were positively associated with QoL only among female participants from South Africa.

## Discussion

Based on data from nationally representative samples of five LMICs, the results of our multivariate analysis showed that male participants generally reported a better QoL than female participants across all five countries. These results show that gender inequality regarding QoL exists and that gender may play a critical role in QoL among older adults in LMICs. Previous studies have argued that men and women are exposed to different cultural norms and social factors [24]. Female participants’ overall social status was lower than that of their male counterparts and they were likely to have a more limited income, more barriers concerning access health care, and more responsibilities regarding household chores. All of these factors could affect their perceived QoL.

Participants from the two African countries—Ghana and South Africa—reported a lower QoL than those in other regions, while participants from China reported the highest QoL among the five countries. The low QoL in African countries may be related to the relatively lower socioeconomic development in these regions. We calculated each country’s mean Gross Domestic Product (GDP) scores between 2007 and 2010 from World Bank data; the GDP rankings of both South Africa and Ghana were relatively low, while China’s GDP ranking was the highest among the five countries. The higher QoL in China could be partly attributed to the country’s rapid increase in living standards and the expansion of medical and pension programs in the country, even though the coverage of the medical program is limited and the pension amount is very low for rural residents [25]. This finding supports the supposition that individual income is the biggest influencing factor on QoL in both male and female participants across all five countries. Although QoL is multidimensional, economic status is a substantial component of QoL, particularly in LMICs.

Health-related variables—such as the presence of comorbidities like arthritis and diabetes and physical function—had a significant influence on QoL for both men and women in virtually all the countries. A number of existing works have reported that health status is closely related to QoL [26, 27]. Specifically, chronic conditions—such as arthritis and diabetes—have a significant impact on an individual’s daily life, as they require management and monitoring of their symptoms. Similar to chronic conditions, physical dysfunction that leads to impaired daily living functions would inevitably affect people’s independence. Therefore, chronic conditions and physical function impairment affect individuals’ sense of well-being and QoL. In countries with limited support from family members and community services, the impact of an individual’s health status would be stronger. The results of this study showed that QoL differed significantly according to participants’ level of cognitive function in community-dwelling older adults. These findings are consistent with the findings of previous studies conducted in developed countries [28, 29].

Health insurance was shown to be related to higher QoL for the general study group but our subgroup analyses showed that health insurance had a significant effect on QoL only among female older adults from Ghana. Our exploratory analyses showed that Ghanaian female older adults with health insurance had both a higher level of education and a higher income (results not shown). Prior research confirms that women with health insurance are more likely to access health care services in Ghana [30] and that they also have fewer out of pocket expenses, as health insurance coverage is good [31]. Since most older adults in China and Russia have health insurance, health insurance may not be an influencing factor on QoL in these countries.

QoL influencing factors were also different by gender. For male participants, social cohesion was significantly associated with higher QoL. However, family relationship (e.g., marital status or living arrangements) was not. This may reflect that male older adults perceive social relationships as more important than family relationships. In the past, men were traditionally associated with socioeconomic activities, as men were deemed to be in charge of the economy, while women were considered to fill a kin keeper role within the family. Because women are perceived to play a more important role in the family, family relationships could be considered to be more important for women than for men.

Being married was associated with a higher QoL among female older adults, except for those in Ghana. In countries such as Ghana, most older women may rely heavily on their husband for financial support and other instrumental support. Therefore, being married more likely indicates financial security and better overall socioeconomic status in females, which in turn, leads to higher QoL. According to the work of Arthur (2006), the average family size in Ghana was larger than that of most other tropical African countries [32] as well as other LMICs. A large family may be a burden for women in Ghana, as women are the main caregivers in their families.

Only in China did community support have a significant effect on QoL among both male and female older adults. The current community-based social welfare programs in China are designed to provide support to individuals with disabilities and to those who do not own property [33]. The exploratory analyses in this study showed that participants receiving community support were more likely to be in the lowest income quintile, have a lower than primary school education, or have more functional limitations. Therefore, it is likely that the participants who reported receiving support from the community had either low socioeconomic status or functional limitations. This may partially explain the negative association between community support and QoL among the Chinese participants.

## Conclusion

Despite its significant results, there are several limitations to this study that need to be considered. First, the study design is cross-sectional and therefore, we were not able to prove causal relationships between the variables. Second, the measures for each variable in the SAGE dataset might not be optimal. Third, missing values differed across the five countries but this was not critical, as less than 15% of all the values were missing across all the covariates and a multiple imputation method was used to address the missing data issue. This study aimed to provide empirical evidence concerning the factors that influence QoL in each separate country and across all LMICs. In this regard, the study provides significant findings. Fourth, we chose 50 years as a cut-off point for older age. Although older age is generally defined at 60 or 65 years in high-resourced countries, older adults may be defined as those over 50 years in low-resourced countries [34] because participants in SAGE countries lived in low-and middle-income countries. Additionally, we did sensitivity analysis after changing the definition of older age at 55 or 60 years; the statistically significant influencing factors were similar.

This study provides critical knowledge that improves our understanding of QoL in LMICs. As one of the main findings, women consistently reported poorer QoL than their male counterparts. These gender disparities in QoL suggests that more—and more effective—policies, programs, and services are necessary to address QoL-related gender equality in these countries. Furthermore, this study showed that income is the biggest influencing factor on QoL. Socioeconomic development—such as improved living standards and social welfare programs—is essential to improve QoL among both male and female community-dwelling older adults in LMICs.

## Data Availability

The datasets generated and analysed during the current study are available on reasonable request, from the WHO Multi-Country Studies Data Archive repository (http://apps.who.int/healthinfo/systems/surveydata/index.php/catalog/sage).

## Abbreviations

GDP: Gross Domestic Product
LMICs: Low- and middle-income countries
QoL: Quality of life
SAGE: Study on Global AGEing and adult health
WHO: World Health Organization
WHODAS: World Health Organization Disability Assessment Schedule
WHOQoL: World Health Organization’s Quality of Life assessment
